# Unbalance of Se and nutritional status in male infertility

**DOI:** 10.5935/1518-0557.20200067

**Published:** 2021

**Authors:** Luana Mara Silva de Castro Pacheco da Cunha, Maria Yasmin Paz Teixeira, Ana Filomena Camacho Santos Daltro, Sebastião Evangelista Torquato Filho, Renata Carmo de Assis, Roberta Freitas Celedonio, Liliane Viana Pires, Carla Soraya Costa Maia, Maria Izabel Florindo Guedes

**Affiliations:** 1 Nutrition department, State University of Ceará, Fortaleza, CE, Brazil; 2 Reproduction Human Center Evangelista Torquato, Fortaleza, CE, Brazil; 3 Nutrition department, Federal University of Sergipe, Aracaju, SE, Brazil

**Keywords:** male infertility, selenium, oxidative stress, overweight

## Abstract

**Objective:**

To evaluate the selenium status and oxidative stress in male infertility cases selected from a private human reproduction center in the state of Ceará, Brazil.

**Methods:**

The present study had a cross-sectional quantitative approach, carried out between January and October 2013 at a Human Reproduction Center. The studied population was composed of 49 male individuals seen at the clinic, aged between 18 and 60 years. Blood samples were collected to measure serum selenium concentrations, erythrocyte activity and glutathione peroxidase. After medical diagnosis, the participants were divided into fertile and infertile groups. Blood samples were collected for establishing Se concentrations in plasma and erythrocytes, and measurements of the enzymatic activity of glutathione peroxidase in the erythrocytes.

**Result:**

it resulted in 53.1% of fertile men and 46.9% of infertile men. The average age of the fertile group was 34.1 years and the infertile group was 37.3 years. Regarding the assessment of nutritional status, the scatter diagram of the infertility group showed a higher body mass index and waist circumference, showing that this group has a higher risk of global and abdominal obesity compared to the fertile group (*p*<0.0001, respectively). There were similarities between the groups regarding caloric intake, macronutrient and selenium intake.

**Conclusion:**

We can conclude that the serum values of selenium, in excess and in deficiency, can be harmful to male fertility.

## INTRODUCTION

Infertility is defined as the inability of a couple to achieve conception or to bring a pregnancy to term after one year or more of regular, unprotected intercourse, and it has been recognized as a public health problem worldwide (World Health Organization - WHO, 2000; Agarwal *et al*., 2015). It affects 10-15% of couples in reproductive age (Winters & Walsh, 2014). Some studies show that environmental, physiological and genetic factors are related to 25% of male factor infertility due to semen quality, including oxidative stress, which has frequently been associated with the problem (Kolesnikova *et al*., 2015; Aitken, 2016).

Oxidative stress adversely affects sperm function by altering membrane fluidity, permeability and sperm functional competence (Kumar & Singh, 2018; Riaz *et al*., 2016; Aitken *et al*., 2016). Sperm membranes are characterized by relatively greater concentrations of polyunsaturated fatty acids that are extremely sensitive to the attack of reactive oxygen species (ROS) (Treulen *et al*., 2015). Oxidative stress attacks not only the fluidity of the sperm plasma membrane, but also the deoxyribonucleic acid (DNA) integrity in the sperm nucleus, making the sperm unable to fertilize an egg or start a viable pregnancy (Bisht & Dada, 2017; Hashemi *et al*., 2018).

Essential trace minerals such as selenium (Se) not only act as antioxidants but also play vital roles in multiple metabolic processes (Türk *et al*., 2014; Ahsan *et al*., 2014). Se is an essential trace mineral recognized as fundamental for human reproduction, spermatogenesis, promotion of normal testicular development and preserving spermatozoa motility (Adeoye *et al*., 2018).

One of Se’s biochemical functions is linked to the enzyme glutathione peroxidase (GPx), a selenoprotein with an antioxidant function that protects the sperm membrane against oxidative stress. GPx acts as an Se reservoir, which is used in emerging needs. Due to its powerful antioxidant effect, Se in the GPx needs to be better investigated as a nutritional factor for the protection of men's health in relation to male infertility, considering that oxidative stress compromises sperm motility, vitality and function (Qazi *et al*., 2019).

The primary source of Se for men is through food. Thus, the Se present in foodstuff can be influenced by the environment and soils, especially those with a high concentration of Se, which can theoretically produce foods richer in this nutrient, enabling its adequate intake. Based on this, the consumption of this mineral in Brazilian diets varies according to region (Tureck *et al*., 2017).

Since Se is a mineral essential for improving reproductive health and Ceará (state of Brazilian Northeast) has a Se-rich soil, the present study’s goal was to evaluate Se, nutritional status and their association with human reproduction in Fortaleza, Ceará- Brazil.

## MATERIAL AND METHODS

### Study design

The present study was a quantitative cross-sectional approach, carried out between January and October of 2013 at a Human Reproduction Center in Fortaleza, capital of the state of Ceará, Brazil. The studied population consisted of male individuals seen at the clinic, aged between 18 and 60 years without chronic diseases, non-smokers, without the use of vitamin and mineral supplements and hormonal medications. The study was conducted according to the ethical principles and guidelines for the protection of human participants in research, which was approved by the Ethics Committee for research of the State University of Ceará (protocol number 5882212.9.00005534 issued on 27/12/2012).

### Data collection

We collected sociodemographic data to characterize the sample. Weight (kg) and height (m) were measured according to the Frisancho methodology (Frisancho, 1990). We calculated the Body Mass Index (BMI) by dividing the body weight by height (kg/m^2^). Nutritional diagnosis was assessed according to the classification for adults (WHO, 2000). Waist circumference (WC) was measured using a flexible, tension-sensitive, non-stretchable measuring tape (Gulick II) placed directly onto the skin. Measurements were taken from the midpoint between the iliac crest and the lowest rib (WHO, 2011).

We assessed dietary intake using the 24-h recall method. Three non-consecutive 24-h dietary recalls were collected, which included two weekdays and one weekend day (Fisberg *et al*., 2005). We used the computer software NutWin (version 2.5, 2002) (fed with the USDA database); the UNIFESP-EPM was used for food intake calculation, with adequacy established by the Dietary Reference Intakes (DRIs) (Institute of Medicine - IOM, 2005). The amounts indicated by the subjects were transformed into homemade measures and analyzed (Pinheiro *et al*., 2008). The Se values were adjusted for energy (IOM, 2000).

Semen analysis was completed by an accredited laboratory according to the standards set by the WHO (2010), which consisted of the macroscopic and microscopic evaluations of the semen.

After medical diagnosis, the participants were divided into fertile and infertile groups. Blood samples were collected for the determination of Se concentrations in plasma and erythrocytes, and we measured their glutathione peroxidase (GPx) enzymatic activity in erythrocytes.

The plasma and erythrocyte Se levels were determined by atomic absorption spectrometry using the hydride generation coupled to the quartz cell HITACHI (Z-5000 model), adapted from Martens *et al*. (2015). GPx activity in erythrocyte was assessed using the kinetic method described by Paglia & Valentine (1967).

We used the commercially available kit (RANSEL 505 - RANDOX Laboratories Ltd, UK - reference range: 27.5 to 73.6 U/g Hb) according to the manufacturer's instructions.

### Statistical analysis

We processed the data using the free software R (version 2.15.0). To compare the mean parameters of the fertile and the infertile groups, we used the Student t-test and ANOVA, and in both cases, we obtained the p-value by the exact test via Bootstrap, ensuring the robustness against the lack of normality. To correlate dietary intake of Se and dietary energy, we used the Kolmogorov - Smirnov test and applied the Pearson’s correlation to assess whether there was a statistically significant correlation between energy and Se. After that, we ran a linear regression analysis ​​between the energy (independent variable) and Se (dependent variable) to make the adjustment for energy. All analyses were considered statistically significant if *p*<0.05.

## RESULTS

We assessed the fertility of 50 male subjects. One participant left the study, yielding 53.1% fertile men and 46.9% infertile men. The mean age of the fertile group was 34.1 (7.6) years and the infertile group was 37.3 (8.1) years.

**Figure 1:**
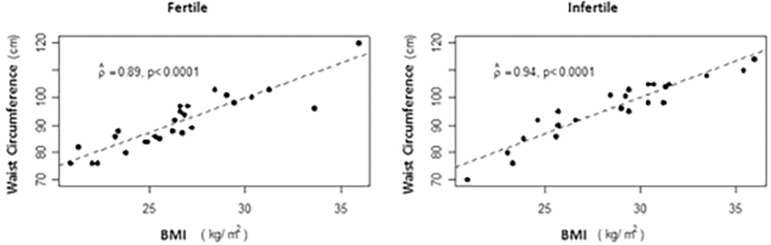
Scatter plot between waist circumference (cm) and body mass intake (Kg/m^2^) by group

Regarding nutritional status assessment, the scatter diagram of the infertility group showed higher BMI and WC, showing that this group presents a higher risk of global and abdominal obesity as compared to the fertile group (*p*<0.0001, respectively) (Table 1) (Figure 1). There were similarities between the groups regarding caloric intake and that of macronutrients and Se (Table 1).

There was no significant difference for serum Se (Table 1). According to Van Dael & Deelstra (1993), the plasma values should be in the range of 60 to 120 µg/L. In this sense, 42.31% of the fertile and 56.52% of the infertile were within this range. In the infertile group, the mean selenium concentration in erythrocytes was higher than in the fertile group (Table 1). According to Van Dael & Deelstra (1993), the reference value of the erythrocyte Se should be in the range of 90-190 µg/L. We had 42.31% of the subjects in the fertile group and 56.52% of the infertile group within this range.

**Figure 2:**
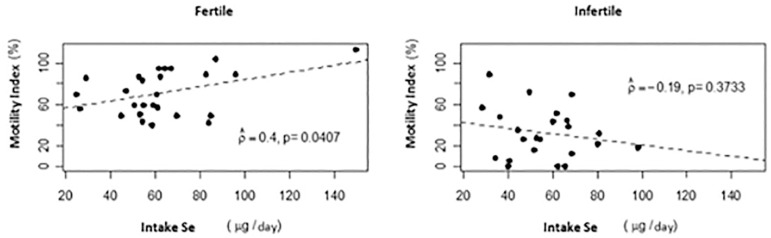
Scatter plot between motility index (%) and intake Se (µg/day) by group

**Figure 3:**
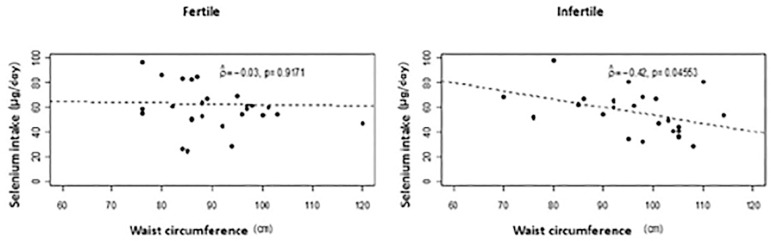
Scatter plot between selenium intake (µg/day) and waist circumference (cm) by group

There was a positive correlation between Se intake and motility index (%) (*p*=0.0407) at the 5% level. The higher the intake of Se, the higher the motility index (%) in the fertile group. (Figure 2). However, there was a negative correlation between WC and Se intake (g/day) in the infertile group (r=-0, 42 *p*=0.0456) (Figure 3).

**Figure 4:**
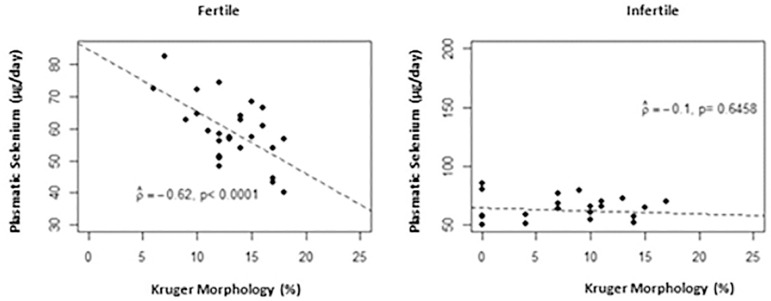
Scatter plot between plasmatic selenium (µg/day) and Kruger morphology (%) by group

On the other hand, there was a significant negative linear correlation (*p*<0.0001) between the Kruger morphology (%) and serum Se in the fertile group. Thus, the higher the plasma Se levels in this group, the lower the Kruger morphology (%) (Figure 4).

**Figure 5:**
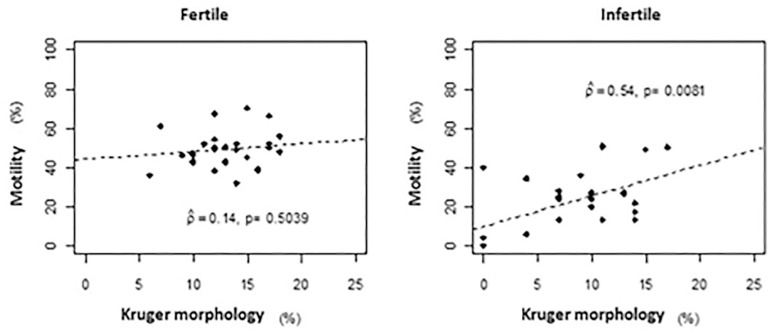
Scatter plot between motility (%) and Kruger morphology (%) by group

Another result of the Kruger's strict sperm morphology (%) showed a significant positive linear correlation (*p*=0.0081), at the 5% level, with the motility index (%) in infertile men (Figure 5).

**Figure 6:**
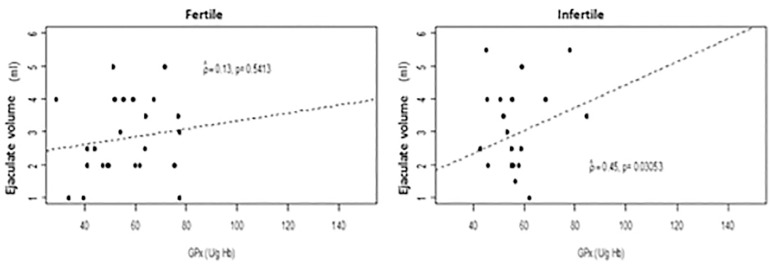
Scatter plot between ejaculate volume (ml) and GPx (Ug/Hb) by group

Between groups there was no difference in GPx activity (Table 1). In addition, there was a positive correlation (*p*=0.0305) between ejaculate volume and GPx in the infertile group (Figure 6). 

**Table 1:** Anthropometric, dietary, biochemical analyses and antioxidants enzymes of the study subjects (Mean values and standard deviations)

	Fertile (n=26)		Infertile (n=23)		*p*
	Mean	SD	Mean	SD	
**Subject characteristics**					
Waist circumference (cm)	90.88	10.16	96.02**	11.07	**<0.0001**
Body mass index (kg/m^2^)	26.48	3.61	28.48**	3.97	**<0.0001**
**Food and selenium intake**					
Energy (kcal/day)	3259.59	1092.40	3126.20	927.81	0.6494
Protein (kcal/day)	18.55	3.13	20.10	6.05	0.2287
Carbohydrate (kcal/day)	48.38	7.18	47.25	8.43	0.5304
Lipid (kcal/day)	32.44	6.53	32.20	5.89	0.8939
Selenium (µg/day)	62.91	25.06	56.08	17.40	0.2795
**Biochemical analysis (µg/L)**					
Plasmatic selenium	59.26	10.02	62.46	12.34	0.3206
Erythrocyte selenium	87.75	48.73	101.75*	71.12	**0.0301**
**Antioxidants enzymes (U/g Hb)**					
Erythrocyte GPx	55.88	13.49	61.45	20.92	0.5144

GPx: Glutathione Peroxidase; SD: standard deviation; Bold values signify that significance level 0.05 was used for statistical interference.

* *p*<0.05;

** *p*<0.01

## DISCUSSION

Selenium biomarkers were associated with male fertility because among fertile men, serum Se levels was inversely associated with the Kruger morphology rate (%), and among the infertile, erythrocyte Se was at higher levels, revealing that serum selenium imbalance, both excess and reduction influence male fertility. GPx activity, also called GPx1, was preserved in both groups without statistical difference between them. Fertile and infertile men were studied for Se concentrations (plasm and erythrocyte).

Trace elements such as selenium are involved in many vital reproductive health and performance processes. However, studies are scarce on the concentration of Se in various body fluids/tissues for optimal reproductive performance. Adequate concentrations of this mineral in the reproductive tissues are fundamental for several processes associated with fertility such as spermatogenesis, maturation and motility of spermatozoids, besides maintenance and improvement of GPx antioxidant activity. Some selenoenzymes such as selenoprotein P (SelP) and the GPx family (especially the GPx 4 isoform) are involved in fertility and reproductive processes (Qazi *et al*., 2019). Thomson (2004) stated that the consumption of 45-50 µg/day is able to optimize the activity of GPx and SelP. Thus, considering the consumption from both groups in this study we can justify the appropriate activity of the GPx enzyme in these groups (Thomson, 2004).

The selenium from food is rich in organic forms of this mineral which has been related to better assimilation being more bioavailable and less toxic. Vitamin B_6_ also participates in the optimization of the GPx system from organic forms of selenium. Therefore, proper nutrition can contribute to fertility. Consumption and adequate concentrations of selenium are linked to testosterone biosynthesis whose mechanism is related to the expression and antioxidant action of SelP in the cells of Leydig and cytosolic GPx acting on the neutralization of H_2_O_2_ produced in this biosynthesis (Qazi *et al*., 2019).

Se deficient and Se excess diets have been reported to result in reduced spermatozoa motility, concentration, and fertility. Diets excessive or deficient in Se affect the gross as well as histological morphology of the testis. Sperm morphology is considered one of the most important seminal parameters, being the best indicator of male fertility. At appropriate levels of serum Se, sperm morphology presents good quality, but as there is inadequacy in consumption, that is, it exceeds the recommended value, this morphology becomes impaired. Therefore, although sperm morphology is a controversial parameter, it is an important result regarding the fertility of men who underwent antioxidant treatment (Qazi *et al*., 2019).

Studies observed by Riaz *et al*. (2016) and Lovercamp *et al*. (2013) show that high levels of plasma Se resulted in a sperm morphology reduction and consequently its motility in fertile men. Our results are consistent with the findings and the same effect was observed in the fertile group. According to WHO (2010), the strict Kruger morphology criteria has a directly association to motility. In the infertile group, the mean erythrocyte Se value was higher than the fertile.

These data suggest that elevated levels of erythrocyte Se decrease motility because the effect of motile onset was probably mediated by secretions of accessory sex glands or during sperm maturation or ejaculation, since there was little evidence of toxic effect upon ingestion (Dorostghoal *et al*., 2017).

Although no difference in food intake was found between groups analysed, the fertility group showed a positive correlation between Se intake and sperm motility (%). These results are in agreement with Foresta *et al*. (2002), Klein (2004), Moslemi & Tavanbakhsh (2011) that showed the relationship between Se ingestion and increased sperm motility.

Besides that, there was a positive correlation between ejaculate volume and GPx in a fertile group. This is in accordance with the study of Türk *et al*. (2014) confirming the presence of GPx in the ejaculate mainly coming from the testes and epididymis, thus regulated by the ingestion of Se.

Thus, decreased GPx activity during Se deficiency and increased lipid peroxidation, indicate increased levels of free radicals which disrupts the normal spermatogenic process and might be responsible for reduced sperm motility and viability leading to reduced fertility and fewer litters. The GPx is responsible for over 75% of antioxidant activity in spermatozoa. In Se excess, increased lipid peroxidation could be attributed to the ability of selenite to form a highly reactive species, a selenopersulfide which generates free radical, superoxide as well as other Reactive Oxygen Species (Shalini & Bansal, 2007; Shalini & Bansal, 2008).

Besides these aspects, it can be verified that the nutritional status indicated higher levels of BMI and WC in the infertile group. Obesity seems to have a deleterious effect on semen quality by changes in epididymal function, reducing the biomarkers of normal accessory gland function. In addition, the ejaculated volume (mL), sperm concentration (million / mL), and total sperm count (millions / ejaculate) are inversely correlated with BMI. Obese men were 19 times more likely to have a lower total sperm count (millions / ejaculate) than eutrophic (Bieniek *et al*., 2016; Oliveira *et al*., 2017; Wang *et al*., 2017; Liu & Ding, 2017). The results are similar to studies designed by Borges Jr. *et al*. (2015) and Schlichthorst *et al*. (2016) that showed a relationship of obesity and infertility with men with mean age of 37 years. The increased WC is associated with obesity-related health risk that can reduce male fertility (Hayden *et al*., 2018; Kasum *et al*., 2016). Se may be a protective factor in male fertility, since the higher the Se intake, the smaller the waist circumference is in the infertile group, and higher the sperm motility index in the fertile group.

Our study demonstrates the possible interferences of obesity, deficiency and excess selenium in male fertility. Thus the action of dietary selenium appears to be U-shaped with respect to reproductive capacity and its markers. Further studies are needed to clarify the mechanisms involved in this process. It also makes an important contribution by calling attention to the use of supplements with Se megadoses present in antioxidant treatments.

## CONCLUSION

From the above findings, we can conclude that fertile and infertile men were suitable for Se concentrations. Serum values of Se, both excess and deficiency can be detrimental to male fertility. In the fertile group, Se consumption was positively correlated with sperm motility and, in the infertile group, negatively correlated with WC.
